# Mitral valve E-point to septal separation (EPSS) measurement by cardiac magnetic resonance imaging as a quantitative surrogate of left ventricular ejection fraction (LVEF)

**DOI:** 10.1186/1532-429X-17-S1-P390

**Published:** 2015-02-03

**Authors:** Abdalla Elagha

**Affiliations:** 1 Cardiology, Cairo University, Cairo, Egypt; 2 NHLBI, NIH, Bethesda, MD, USA

## Background

Using Echocardiography, the EPSS is a straightforward approach that reflects LV function, but its use has been limited to echocardiography technique, without solid quantitative correlation to LVEF. Also, it may cause underestimation of EF due to endocardial echo dropout. Cardiac MRI has a better spatial resolution than echocardiography, and is characterized by superior endocardial border definition, facilitating more accurate assessment of structural borders. The MRI LVEF by Simpson's method is widely considered the most accurate and most reliable method for quantifying the LVEF. Assessment of EPSS by CMRI seems very attractive and simple measurement, which can be an additional standard tool in clinical MRI report for quantitative evaluation of LV function.

## Methods

We studied a total of 143 patients, who underwent complete CMR study. Nineteen patients with significant aortic insufficiency, mitral stenosis or prosthesis, and septal hypertrophy were excluded. Short-axis cross-sectional stack images were used to estimate LVEF by Simpson's method. The EPSS was determined using image plane corresponding to a 3-chamber view, known as LVOT view. The EPSS was measured in millimeters (mm) as the minimal separation distance between the mitral valve anterior leaflet and the ventricular septum, usually occurring at the maximal filling phase of cardiac cycle. Cautious tracking of the leaflet through diastole, frame by frame, allows measurement of shortest distance between leaflet tip and the interventricular septum. Furthermore, we divided patients into two groups according to presence or absence of fibrosis on delayed hyperenhancement MRI study.

## Results

The LVEF ranged from 12-79 %. The EPSS ranged from 2.2-26.1 mm. We used correlation and linear regression analysis to analyze the relation between the LVEF and the EPSS. Correlation coefficient revealed to be very strong (r= -0.92; 95% Confidence interval for r= -0.95 to -0.87) with high significant level (P<0.0001). Using regression analysis, MRI LVEF could be estimated from the following equation (LVEF = 78.1569 - (2.6661 x EPSS)), with strong regression coefficient (r^2^= 0.86).

Also, correlation and regression coefficients were found to be closely similar in both groups with and without DHE fibrosis (r= -0.94 with no fibrosis, r= -0.91 with fibrosis; P<0.0001 for both groups).

## Conclusions

EPSS measurement by cardiac MRI is an easy and accurate method, which allows parallel assessment of LV function, in patient with and without myocardial fibrosis. The EPSS can generate a rapid quantitative idea on LV function, especially when acquirement of multiple breath-hold short-axis images is difficult.

## Funding

No disclosure.

**Figure 1:**
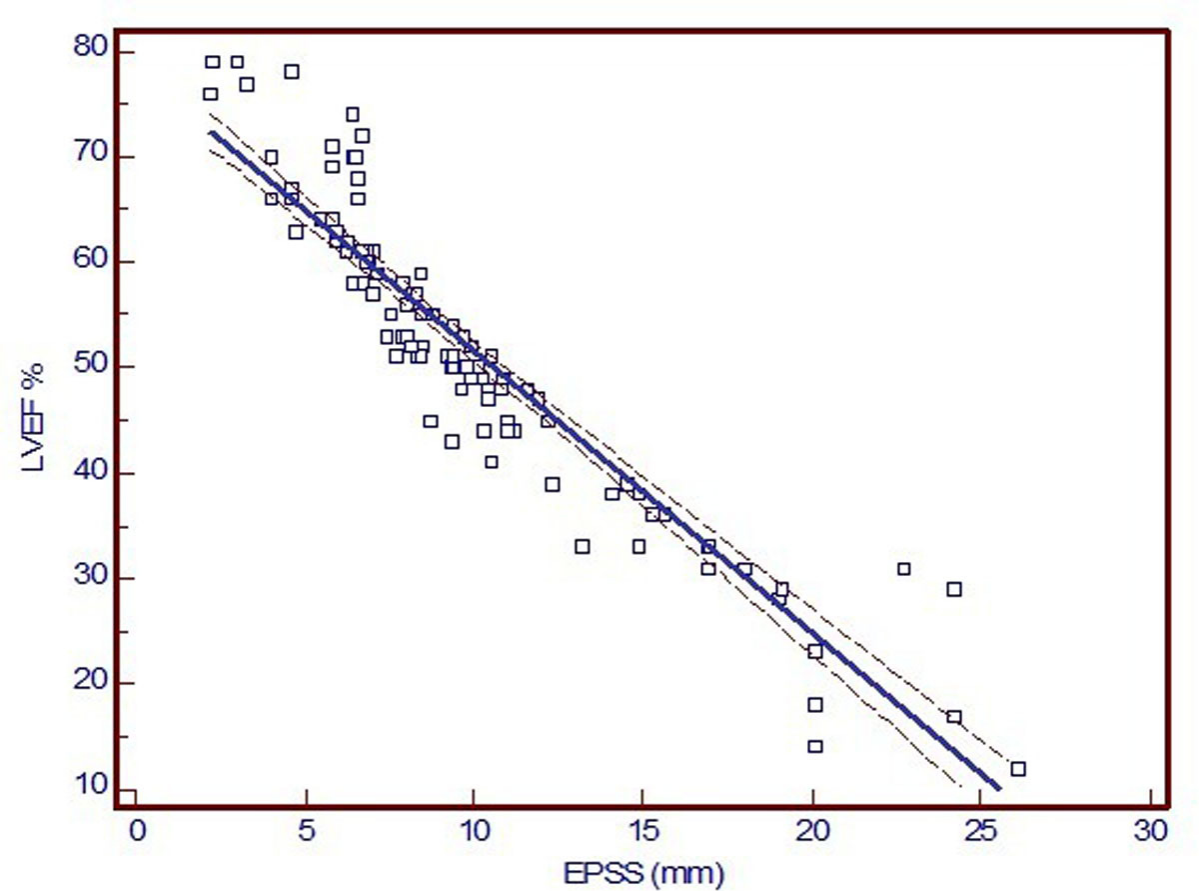
Scatter diagram of the MRI LVEF vs.the EPSS with regression line (solid line) and 95% confidence intervals (dotted lines).

**Figure 2:**
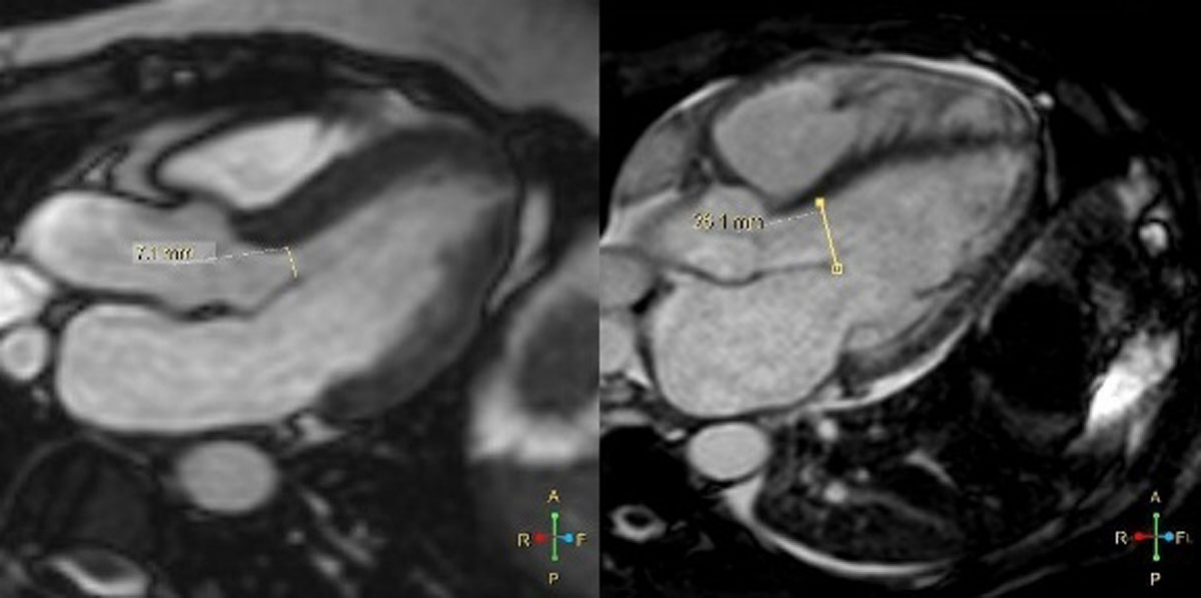
**Measurement of EPSS in 3-chamber view (cine SSFP image).** The mitral valve E-septal separation measured 7.1mm (left) and 26.1 mm (right), as shown by the line representing the minimal separation distance between the mitral valve anterior leaflet tip and the ventricular septum. The E.F. was 59 % and 12%; respectively

